# Predictive value of S100B and brain derived neurotrophic factor for radiofrequency treatment of lumbar disc prolapse

**DOI:** 10.1186/s12871-024-02527-4

**Published:** 2024-04-26

**Authors:** Wael Fathy, Mona Hussein, Rehab Magdy, Hatem Elmoutaz, Neveen A Youssef, Marwa F Abd Alla, Ahmed M El Shaarawy, Mohamed Abdelbadie

**Affiliations:** 1 Department of Anesthesiology, Surgical ICU and Pain Management, Beni-Suef University, Salah Salem Street, Beni-Suef, 62511 Egypt; 2 Department of Neurology, Beni-Suef University, Beni-Suef, Egypt; 3 Department of Neurology, Cairo University, Cairo, Egypt; 4 Department of Clinical and Chemical Pathology, Beni-Suef University, Beni-Suef, Egypt; 5 Department of Medical Biochemistry and Molecular Biology, Beni Suef University, Beni-Suef, Egypt

**Keywords:** Lumbar disc prolapse, Pulsed radiofrequency, NRS, FRI, S100B, BDNF

## Abstract

**Background:**

This work aimed to analyze serum S100B levels and brain-derived neurotrophic factor (BDNF) in patients with lumbar disc prolapse to test their predictive values concerning the therapeutic efficacy of pulsed radiofrequency.

**Methods:**

This prospective interventional study was carried out on 50 patients candidates for radiofrequency for treating symptomatic lumbar disc prolapse. Pain severity and functional disability were assessed using the Numeric Rating Scale (NRS) and Functional rating index (FRI) before as well as two weeks, 1, 3, and 6 months after the radiofrequency. Quantitative assessment of serum S100B level and BDNF was done for all the included patients one day before radiofrequency.

**Results:**

The scores of NRS and FRI were significantly improved at two weeks, 1, 3, and 6 months following radiofrequency (P-value < 0.001 in all comparisons). Statistically significant positive correlations were found between duration of pain, NRS, and S100B serum level before radiofrequency, and both NRS (P-value = 0.001, 0.035, < 0.001 respectively) and FRI (P-value = < 0.001, 0.009, 0.001 respectively) 6 months following radiofrequency. Whereas there were statistically significant negative correlations between BDNF serum level before radiofrequency and both NRS and FRI 6 months following radiofrequency (P-value = 0.022, 0.041 respectively). NRS and S100B serum levels before radiofrequency were found to be independent predictors of NRS 6 months following radiofrequency (P-value = 0.040. <0.001, respectively).

**Conclusion:**

Serum level of S100B is a promising biomarker that can predict functional outcomes after pulsed radiofrequency in patients with lumbar disc prolapse.

## Introduction

Low back pain is one of the most prevalent complaints, as approximately 80% of the population experience at least one episode of low back pain during their lifetime. Within the vast etiologies of low back pain, the most common cause is lumbar disc prolapse which results from intervertebral disc degeneration and herniation [1]. Pain in lumbar disc prolapse is a significant problem contributing to considerable morbidity and health care costs [2, 3]. It is primarily supposed that some inflammatory mediators mediate radicular pain along with the mechanical factor [2]. Pulsed radiofrequency is a minimally invasive therapeutic procedure widely used due to its low complication rate and favourable outcome [4]. It acts through neuromodulation rather than causing thermal ablation when applied to the dorsal root ganglia [5]. Earlier studies mainly focused on investigating clinical factors as potential predictors of the therapeutic success of pulsed radiofrequency [6]. Yet, molecular biomarkers received less attention in this field.

Recently, activated astrocytes might also be relevant for the pathophysiology of neuroinflammation and pain signaling [7, 8]. The S-100B is a Ca2+-binding protein primarily secreted by astrocytic glial cells of the central nervous system (CNS). It is a well-established marker of traumatic brain injury (TBI), where elevated levels indicate the severity of the injury [9, 10]. It has been reported that S-100B might be a promising serum biomarker to predict the functional outcome of surgical decompression in symptomatic spinal cord compression [11]. However, it is not well-defined whether it contributes to pain-related disc prolapse [12].

In addition to the glial-derived neurotrophic protein (S100β), several lines of evidence point out that the brain-derived neurotrophic factor (BDNF) is involved in the pain conception, control, and regeneration of nerve injury [13]. BDNF is also induced in dorsal root ganglia (DRG) neurons after nerve injury and can be released from primary afferents in the spinal cord [14]. Strong evidence suggests that peripheral neuronal damage induces BDNF expression at the site of the damage, which promotes the regeneration of damaged neurons [15].

Indeed, exploring reliable molecular biomarkers such as S100B or BDNF as prognostic and predictive factors for the outcome of pulsed radiofrequency would significantly impact clinical decisions as it would help in selecting the patients who are expected to gain benefit from radiofrequency.

Accordingly, this work aimed to analyze serum S100B levels and BDNF in patients with lumbar disc prolapse to test their predictive values concerning the therapeutic efficacy of pulsed radiofrequency.

## Methods

### Study design and eligibility criteria

In this prospective interventional study, 50 patients candidates for radiofrequency were evaluated for treating symptomatic lumbar disc prolapse. They were recruited from the Pain Centre in Beni-Suef governorate and the Neurology clinic in Beni-Suef University Hospital from January 2022 to December 2022. The study was registered in ClinicalTrials.gov on 18/1/2022 (the identification number is NCT05193461).

Eligibility criteria: poor response to conservative pharmacological treatment and physiotherapy in patients with more than three months of lumbar disc prolapse.

We excluded patients with other medical disorders that might account for their pain-related disability such as those with radiological evidence of hip or facet osteoarthritis, sacroiliitis, paravertebral abscess, Pott’s disease, osteomyelitis or neoplastic lesion affecting the vertebrae. Patients candidates for surgery who had severe lumbar disc herniation affecting sphincteric or motor functions, patients having contraindications to radiofrequency (sepsis or coagulopathy), and pregnant patients were also excluded.

**Fig. 1:**
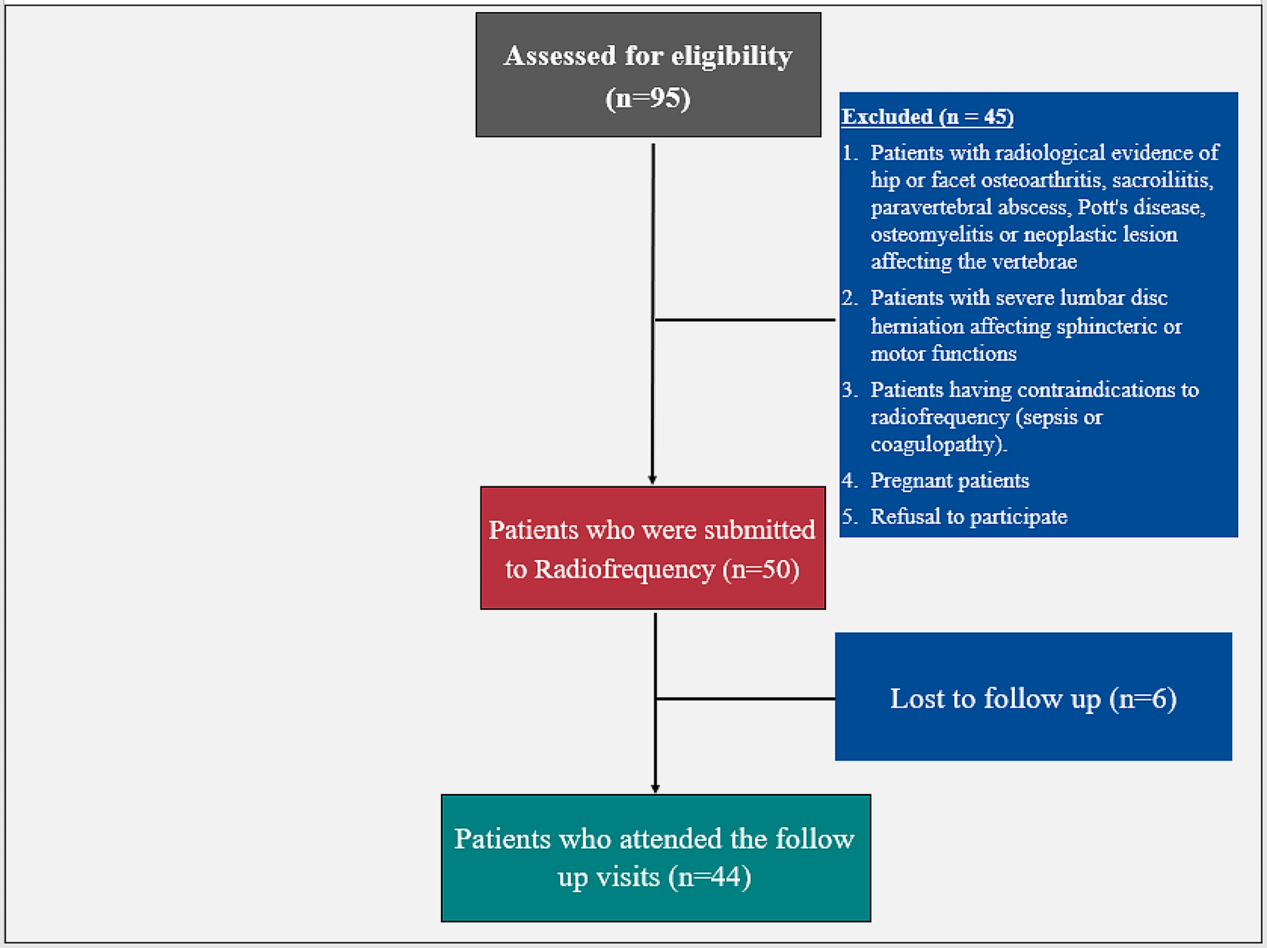
Flow diagram for the included and excluded patients

Regarding the flow of the participants through the study, out of 95 patients assessed for eligibility, forty-five cases were omitted (38 didn’t fulfill the inclusion criteria, and seven refused to participate). Fifty patients received the allocated intervention, and six were lost to follow-up (Fig. 1).

### Clinical assessment

The included patients were subjected to detailed history and neurological assessment to confirm the diagnosis of lumbar disc prolapse.

Pain severity and functional disability were assessed using the Numeric Rating Scale (NRS) and Functional rating index (FRI) by a skilled neurologist before as well as two weeks, 1, 3, and 6 months after the radiofrequency.

NRS is an 11-point numeric scale used to assess pain severity (0–10), where 10 indicates the worst possible pain, and 0 indicates no pain [16].

FRI was used to assess functional disability, including ten items; two concerning pain intensity and eight concerning daily activities. Each item scored from 0 to 4 (no pain to severe pain). The total score ranged from 0 to 100% (no evidence of disability to severe disability) [17].

### Interventional pain procedure

The included patients were requested to stop any medications used for the lumbar disc prolapse two days before the interventional pain procedure. At the time of radiofrequency, the selected patient was brought to the operating room and connected to a monitor. The selected patient was placed in a prone position, and a sterile drape was put over the involved back area. Fluoroscopic guidance was used to identify the affected spinal segment(s) and direct the pulsed RF to the targeted DRG in the intervertebral foramen. Pulsed RF offers the advantage of pain control without the tissue destruction and painful sequelae associated with conventional continuous RF. The used device was the Neurotherm NT2000iX. It delivers electrical current in very brief pulses. The recommended protocol involves delivering a current of 50,000 Hz in 20-millisecond pulses, at a frequency of 2 per second. The relatively long pause between pulses allows any heat generated to dissipate and thus prevent the development of any thermal lesion. This will alter the transmission of pain signals in the nerve without creating a histological lesion [18].

Minimal sedation was done using 0.02 mg/kg midazolam intravenously. 1% lidocaine was infiltrated into the skin at the site of injection. RF needle (22 G, 10 cm, curved, with a 10 mm active tip) was inserted into the targeted location, and the position was verified using fluoroscopic lateral and AP views. The final definite position of the radiofrequency probe was determined by sensory stimulation (50 Hz), producing numbness in the radicular pain distribution. Motor stimulation was done at 2 Hz to avoid injury of the anterior nerve root. Pulsed RF was performed at 42 °C for 120 s twice. After RF lesioning, we confirmed the location inside the epidural space by examining the spread pattern of contrast. Then, we injected a total dose of 80 mg of methylprednisolone acetate with 2 ml of 1% lidocaine. After the procedure, the patient is monitored and managed for any possible procedural complications such as neural trauma, dysesthesia, paresthesia, or haematoma formation.

### Laboratory assessment

#### Quantitative determination of serum S100B level

It was done one day before the radiofrequency procedure by applying a sandwich enzyme-linked immunosorbent assay (ELISA) (MyBioSource com. Catalog No: MBS3503148). We carried out the assay at the clinical pathology department on an automated ELISA platform at Beni-suef University hospital.5mL of venous blood was collected from the participants in a plain tube. Centrifugation of blood samples at 1000 rpm speed for 15 min was done. The supernatant serum was collected and stored at -20 C for the assay. We applied 100 µL of standard or serum samples into each well, then incubated at 37 C. for 90 min. The liquid was removed, 100µL Biotinylated Detection Ab was added to each well and incubated for 60 min at 37 C. The wells were washed four times with a wash solution (Phosphate _ Buffered Saline, PBS, pH 7.4). We added 100 µL of Avidin-horseradish peroxidase (HRP) conjugate to each well and incubated for 30 min at 37 C. Each well was washed five times by adding a Washing Solution. We applied 90µL of Substrate Reagent to each well, covered and incubated for 15 min at 37 C. The last step, We added 50 µL of Stop Solution to each well and read the optical density at 450 nm within 5 min.

#### Validity of the ELISA techniques for S100B

This kit recognizes Human S100B in samples. No significant cross-reactivity or interference between Human S100B and analogues was observed.

#### Reliability

Coefficient of variation (CV) is < 10%. Intra-assay Precision (Precision within an assay): 3 samples with low, mid range and high level Human S100B were tested 20 times on one plate, respectively. Inter-assay Precision (Precision between assays): 3 samples with low, mid range and high level Human S100B were tested on 3 different plates, 20 replicates in each plate.

#### Quantitative determination of serum BDNF level

It was done one day before the radiofrequency procedure by applying a sandwich ELISA technique (MyBioSource com. Catalog No: MBS700602). We carried out the assay at the clinical pathology department on an automated ELISA platform at Beni-suef University Hospital. 5 mL of venous blood was collected from the participants in a plain tube. Centrifugation at 1000 rpm speed for 15 min was done. The supernatant serum was collected and stored at -20 C for the assay. We applied 100 µL of standard or serum samples into each well, then incubated for two hours at 37 C. The liquid was removed, added 100µL Biotinylated Ab to each well. Incubation for 60 min at 37 C. was done. Washing The wells three times with a wash Solution. We applied 100µL of Avidin-horseradish peroxidase (HRP) conjugate to each well and incubated for one hour at 37 C. Each well was washed five times by adding a Washing Solution. We applied 90µL of TMB Substrate to each well, covered and incubated it for 15 min at 37 C. The last step, We added 50 µL of Stop Solution to each well and read the optical density at 450 nm within 5 min.

#### Validity of the ELISA techniques for BDNF

This assay has high sensitivity and excellent specificity for detection of human BDNF. No significant cross-reactivity or interference between human BDNF and analogues was observed.

#### Reliability

Intra-assay Precision CV%<8% and Inter-assay Precision CV%<10%.

#### Outcomes of the study

The primary outcome was to investigate the potential role of S100B and BDNF in predicting outcomes from using radiofrequency in treating lumbar disc-related radicular pain. Such laboratory biomarkers were known to be potentially impacted in lumbar disc prolapse, so they were expected to have a predictive value of outcome from treatment.

The secondary outcome was to study the role of age, duration of pain, number of prolapsed discs, pain intensity, and functional disability before radiofrequency, in predicting the outcome of using radiofrequency in treating lumbar disc-related radicular pain.

#### Sample size

The sample size was calculated using G*Power version 3.1.9.7 Software based on the provisional results of a pilot study before starting the current study. The probability of type I error (α) was 5%, effect size = 0.463, critical t = 2.011, df = 48, and non-centrality parameter λ = 3.69. So, a sample size of 50 patients was requisite to attain a statistical power (1–β) of 95%.

### Statistical analysis

IBM SPSS Version 25 was used to analyze the data. Categorical variables such as sex and degree of the most prolapsed disc were presented as numbers and percentages. Quantitative variables such as age, duration of pain, number of prolapsed discs, NRS and FRI were presented as mean and standard deviation (SD). Paired sample t-test was used to compare pre-and post-interventional NRS and FRI. Pearson correlation was used to test the relationship between the included patients’ age, clinical, imaging, and laboratory characteristics and both NRS and FRI 6 months after radiofrequency. A multiple linear regression model was done to identify predictors of NRS and FRI 6 months following radiofrequency after being adjusted for their potential mutual confounding effect. Age, duration of pain, number of prolapsed discs, NRS and FRI before radiofrequency, and BDNF and S100B serum levels before radiofrequency were used as the independent variables. P-value ≤ 0.05 was considered statistically significant. All tests were two-tailed.

## Results

### General characteristics of the patients

Fifty patients (29 males and 21 females) diagnosed with symptomatic lumbar disc prolapse and indicated for radiofrequency were prospectively evaluated. The mean value for age was 55.06 (12.65) years, for the duration of pain was 22.32 (13.62) months, for the number of prolapsed discs was 2.76 (1.06), for NRS before radiofrequency was 8.68 (1.13), for FRI before radiofrequency was 72.17 (14.84), for BDNF serum level before radiofrequency was 5.098 (1.34) ng/ml, and for S100B serum level before radiofrequency was 5.3 (1.26) ng/ml (Table 1).

**Table 1:** Demographics, clinical, imaging and laboratory characteristics of the included patients

		Patients (*n* = 50)
Age [Mean (SD)]		55.06 (12.65)
Sex	Males [n (%)]	29 (58%)
	Females [n (%)]	21 (42%)
Duration of pain in months [Mean (SD)]		22.32 (13.62)
Number of prolapsed discs [Mean (SD)]		2.76 (1.06)
Degree of the most prolapsed disc	Bulge [n (%)]	14 (28%)
	Protrusion [n (%)]	32 (64%)
	Herniation [n (%)]	4 (8%)
NRS before radiofrequency [Mean (SD)]		8.68 (1.13)
FRI before radiofrequency [Mean (SD)]		72.17 (14.84)
BDNF serum level before radiofrequency in ng/ml [Mean (SD)]		5.098 (1.34)
S100B serum level before radiofrequency in ng/ml [Mean (SD)]		5.3 (1.26)

BDNF: Brain derived neurotrophic factor, FRI: Functional rating index, NRS: Numeric rating scale

### Pain severity and functional disability before and after radiofrequency

At two weeks, 1, 3, and 6 months following radiofrequency, the scores of NRS and FRI were significantly improved (P-value < 0.001 in all comparisons) (Table 2).

**Table 2:** NRS and FRI before and after radiofrequency

		Patients (*n* = 50)
NRS (Mean (SD)	Before radiofrequency	8.68 (1.13)
	After 2 weeks	5.78 (2.3)
	P- value	< 0.001*
	Before radiofrequency	8.68 (1.13)
	After 1 months	5.44 (2.48)
	P- value	< 0.001*
	Before radiofrequency	8.68 (1.13)
	After 3 months	5.12 (2.97)
	P- value	< 0.001*
	Before radiofrequency	8.68 (1.13)
	After 6 months	5.34 (2.86)
	P- value	< 0.001*
FRI (Mean (SD)	Before radiofrequency	72.17 (14.84)
	After 2 weeks	53.16 (19.01)
	P- value	< 0.001*
	Before radiofrequency	72.17 (14.84)
	After 1 months	45.64 (21.075)
	P- value	< 0.001*
	Before radiofrequency	72.17 (14.84)
	After 3 months	43.16 (22.93)
	P- value	< 0.001*
	Before radiofrequency	72.17 (14.84)
	After 6 months	42.78 (24.16)
	P- value	< 0.001*

FRI: Functional rating index, NRS: Numeric rating scale

*P-value ≤ 0.05 is considered significant

Statistically significant positive correlations were found between duration of pain, NRS, and S100B serum level before radiofrequency, and both NRS (P-value = 0.001, 0.035, < 0.001 respectively) and FRI (P-value = < 0.001, 0.009, 0.001 respectively) 6 months following radiofrequency. Whereas BDNF serum levels before radiofrequency were negatively correlated with both NRS and FRI 6 months following radiofrequency (P-value = 0.022, 0.041 respectively) (Table 3).

**Table 3:** Correlations between age, clinical, imaging and laboratory characteristics of the included patients, and both NRS and FRI 6 months after radiofrequency

	NRS 6 months after radiofrequency		FRI 6 months after radiofrequency	
	(r) coef.	P-value	(r) coef.	P-value
Age	0.140	0.333	0.008	0.954
Duration of pain in months	0.438	0.001*	0.611	< 0.001*
Number of prolapsed discs	-0.194	0.176	-0.147	0.308
NRS before radiofrequency	0.299	0.035*	0.366	0.009*
FRI before radiofrequency	0.014	0.922	0.241	0.092
BDNF serum level before radiofrequency in ng/ml	-0.324	0.022*	-0.291	0.041*
S100B serum level before radiofrequency in ng/ml	0.499	< 0.001*	0.469	0.001*

BDNF: Brain derived neurotrophic factor, FRI: Functional rating index, NRS: Numeric rating scale

*P-value ≤ 0.05 is considered significant

### Predictors of pain severity and functional disability six months after radiofrequency

A multiple linear regression model was done to identify predictors of NRS and FRI 6 months following radiofrequency. Age, duration of pain, number of prolapsed discs, NRS and FRI before radiofrequency, and BDNF and S100B serum levels before radiofrequency were used as the independent variables.

NRS and S100B serum levels before radiofrequency were found to be independent predictors of NRS 6 months following radiofrequency (P-value = 0.040. <0.001, respectively). Also, duration of pain and S100B serum levels before radiofrequency were found to be independent predictors of FRI 6 months following radiofrequency (P-value = 0.002. 0.003, respectively) (Table 4).

**Table 4:** Predictors of NRS and FRI 6 months after radiofrequency

Dependent variables	Independent variables	B	P-value	95% CI		Adjusted R Squared
				Lower bound	Upper bound	
NRS 6 months after radiofrequency	Constant	-8.042	0.098	-17.643	1.559	0.394
	Age	0.043	0.098	-0.008	0.094	
	Duration of pain in months	0.040	0.202	-0.022	0.101	
	Number of prolapsed discs	-0.526	0.135	-1.224	0.172	
	NRS before radiofrequency	0.775	0.040	0.036	1.514	
	FRI before radiofrequency	-0.036	0.116	-0.082	0.009	
	BDNF serum level before radiofrequency in ng/ml	0.181	0.547	-0.421	0.783	
	S100B serum level before radiofrequency in ng/ml	1.237	< 0.001	0.602	1.872	
FRI 6 months after radiofrequency	Constant	-75.21	0.057	-152.737	2.314	0.445
	Age	0.156	0.452	-0.259	0.571	
	Duration of pain in months	0.806	0.002	0.308	1.303	
	Number of prolapsed discs	-1.038	0.712	-6.673	4.597	
	NRS before radiofrequency	3.909	0.193	-2.057	9.875	
	FRI before radiofrequency	0.088	0.634	-0.281	0.457	
	BDNF serum level before radiofrequency in ng/ml	2.265	0.352	-2.596	7.125	
	S100B serum level before radiofrequency in ng/ml	8.021	0.003	2.894	13.148	

*BDNF*: Brain derived neurotrophic factor, FRI: Functional rating index, NRS: Numeric rating scale

*P-value  ≤ 0.05 is considered significant

## Discussion

This study aimed to provide novel insights into S100B and BDNF serum levels in patients with pain-related lumbar disc prolapse for the emerging field of injury-related biomarkers as potential predictors concerning the long-term outcome after pulsed radiofrequency. The most striking finding in the present study is that lowered serum S100B levels before radiofrequency could predict a higher analgesic effect and functional improvement six months after the intervention.

Previous studies obtained similar results confirming the role of S100B serum levels in predicting motor score improvement in patients with spinal cord compression caused by epidural empyema [19] and metastasis [11].

In line with the current results, H Ishiguro, T Kaito, K Hashimoto, J Kushioka, R Okada, H Tsukazaki, J Kodama, Z Bal, Y Ukon, S Takenaka, et al. [20] found that the pharmacological administration of ONO-2506 could attenuate neuropathic pain and improve motor function in a rat model of spinal cord contusion. Such therapeutic efficacy was mediated by inhibiting S100B production, a well-documented biomarker for astrocytic reaction. Another animal study by DG Kwak and DG Lee [21] observed that the increased activity of astrocytes in the dorsal horn returned to its control levels on day 14 following surgery in a rat model of lumbar disc herniation.

Altered S100B level was also documented in other pain conditions, including fibromyalgia [22] and migraine [23]. In both studies, increased serum S100B levels were associated with a decreased pain threshold, supporting the negative correlation found in this study between serum S100B levels and NRS and FRI. Additionally, elevated S100B levels were found in the spinal dorsal horn of HIV patients with chronic pain compared to those without pain [24].

On the other hand, this study showed a significantly negative correlation between serum BDNF levels and NRS and FRI scores six months after the application of radiofrequency. Q Li, Y Liu, Z Chu, J Chen, F Dai, X Zhu, A Hu and C Yun [25] reported an increase in BDNF expression in DRG of rat models of lumbar spinal stenosis that was inversely associated with pain severity and walking distance, consistent with the current results.

Overwhelming evidence points out that BDNF can be substantially expressed by human nucleus pulposus cells, whose levels are up-regulated robustly in response to proinflammatory cytokines, mainly IL-1β and TNFα [26–28]. Then his turn begins to promote neuronal growth and survival, axon growth, synaptic plasticity, and the restoration of nerve injury [29]. Contrariwise, neuropathic pain might be inhibited by the spinal blockade of Truncated BDNF Receptor TrkB.T1 [30]. Yet, serum BDNF level was not found as a predictor of post-interventional improvement in either pain or functional scores in the present study.

Lumbar structures including the intervertebral disc, facet joints, muscles, fascia, ligaments, and joint capsules can largely contribute to the pain mechanism in disc prolapse [31]. Histopathological studies revealed neuromuscular structural changes in these patients evident by infiltration of damaged nerves and dorsal root ganglia by macrophages along with muscular fatty infiltration and structural remodelling within muscle fibers in the form of reduction of type I fibers with an abundance of type II and intermediate IIc fibers were all described in patients with chronic low back pain [32, 33]. Also, it was found that patients with chronic low back pain had higher trigger points in the psoas muscle than healthy subjects. Sustained nociceptive input from such trigger points can drive chronic musculoskeletal pain [34]. Moreover, damaged lumbar intervertebral disc and endplates can lead to the pathologic invasion of nerve growth, followed by the compression of disc prolapse on nerve roots, thereby causing traumatic neuropathic pain [35].

It is well evident that these above-mentioned lumbar structures are rich in nociceptors [31]. Activation of these nociceptors triggers inflammatory biochemical pathways in the form of upregulated expression of IL-1β, TNF-α, and VEGF in degenerated disc tissue, along with enhanced neovascularization, which can directly cause nerve damage accounting for the peripheral sensitization in patients with chronic low back pain [35]. On the other hand, the central mechanism of chronic low back pain involves central excitation of central pain regions including sensorimotor, descending pain modulatory systems, attention and default mode networks. Also, glial cell activation and cytokines induced by central neuroinflammation add to the lowered pain threshold [36].

Interestingly, the interaction between BDNF released from the dorsal root ganglia with TrkB receptors placed on primary afferent nerve endings and post-synaptic tracts in the spinal cord assists in magnifying ascending sensory signals, contributing to the preservation of pain-mediated-central sensitization [37].

The current study indicates that serum levels of S100B may be more representative of neural injury compared to BDNF and thus identify useful reference points to predict therapeutic outcomes. Serum BDNF is principally dependent on the degree of disruption of the blood-brain barrier (BBB) and blood–spinal cord barrier (BSCB), making CSF BDNF levels more informative than the serum samples [38]. On the other hand, the S100B protein is characterized by its low molecular weight, facilitating its emergence into the peripheral circulation during the early phases of BBB or BSCB disruption [12].

The main limitation of this study is the absence of a control group, which might add to the credibility of the results. Second, the a lack of specificity of the studied biomarkers. Despite efforts made by the authors to avert other confounders that might alter serum measurements, some issues remain challenging to rule out. For instance, the serum level of BDNF could be altered by comorbid depression, SSRIs, or SNRIs [39], and underlying hidden neurodegenerative diseases that have not yet manifested [40]. Further research may be worthwhile with multiple time point measurements of the serum S100B and interpreting their levels with other nerve injury-relevant biomarkers (ex, glial fibrillary acidic protein, interleukin-6) for their additive predictive value for pain-reliving effects of other less invasive interventions like dry needling or ultrasound-guided procedures in pain-related disc prolapse. Further research should target studying the predictors of therapeutic efficacy of radiofrequency disc decompression.

## Conclusion

Higher serum levels of S100B and lower serum BDNF were associated with higher pain intensity and functional limitations in patients with lumbar disc prolapse. However, only serum levels of S100B can predict functional outcomes after pulsed radiofrequency in patients with lumbar disc prolapse. A low serum level of S100B can predict post-interventional pain and functional improvement, ultimately assisting in identifying the best candidates for pulsed radiofrequency.

## Data Availability

Authors report that the data and materials that support the results or analyses presented in the current study will be freely available upon request.

## Declarations

The authors report that the content has not been published or submitted for publication elsewhere.

**Ethical approval and consent to participate**.

Written informed written consent was signed by all patients. The study was performed in agreement with the Declaration of Helsinki. Ethical approval was obtained from the Research ethical committee of Beni-Suef University; the approval number was FMBSUREC&002F06042021&002FFathy&002D2.
